# Inverted U-shaped relationship between education and family health: The urban-rural gap in Chinese dual society

**DOI:** 10.3389/fpubh.2022.1071245

**Published:** 2023-01-11

**Authors:** Changli Jia, Yanwen Long, Xiaoxia Luo, Xiao Li, Wenjing Zuo, Yibo Wu

**Affiliations:** ^1^Taikang Medical School (School of Basic Medical Sciences), Wuhan University, Wuhan, China; ^2^School of Public Health, Global Health Institute, Wuhan University, Wuhan, China; ^3^Institute of Education Sciences, Wuhan University, Wuhan, China; ^4^School of Public Health, Peking University, Beijing, China

**Keywords:** family health, education, inverted U-shaped relationship, work-family conflict, urban-rural inequality

## Abstract

**Introduction:**

The Healthy China Initiative emphasizes family health. Education is an upstream determinant of health, which can both achieve upward mobility and cause class solidification.

**Methods:**

Using nationwide large-scale data collected in 2021, the present study explored the relationship between education and family health in the urban-rural dual society via Oaxaca-Blinder decomposition and propensity score matching.

**Results:**

Our data revealed disparities in family health, educational attainment, household income, healthcare coverage, and job type between urban and rural China. An inverted U-shaped relationship existed between increasing years of education and family health. The upper limit was 17.1 years for urban residents and 13.7 years for rural residents, with limited health benefits from higher education obtained by rural residents. Mediated by work-family conflict, highly-educated people received gradually diminishing health returns. The results of the Oaxaca-Blinder decomposition showed that 25.8% of the urban-rural gap in family health could be explained by the disparity in education. Urban residents could translate cultural capital and economic capital into health capital to a greater extent. After propensity score matching, a robust, inverted U-shaped relationship was found between education and family health. The inverted U-shaped relationship was found to replace family health with self-rated health and quality of life.

**Discussion:**

Family-centered public health and education programs, policies, and goals should be developed to break urban-rural dual structure barriers and advance social equity in China.

## 1. Introduction

*The Health China Initiative* aims at narrowing the gap in basic healthcare services between urban and rural areas, regions, and communities, to achieve universal health coverage and social equity. The awareness of healthy life and family health (FH) management has been enhanced in recent years. The family lays the foundation for individual growth and sustainable development (1, 2), which exerts an unparalleled influence and resource for health maintenance and disease prevention (3, 4), especially during the post-coronavirus disease 2019 (COVID-19) era.

The hukou system was first devised in 1955 and propagated in 1958 as a measure of social control to restrict rural populations from access to state-allocated products, welfare, and rights. Based on the place of birth and lineage (i.e., mother's hukou type), each person is assigned a hukou type [either agricultural (rural) hukou or non-agricultural (urban) hukou] (5). Because the hukou system strictly confined people to the land they were born for a few decades, a de jure rural-urban division has been created (6). Due to the hukou system, there are distinct differences in geographical environment, welfare resources, behavioral habits, and cultural values between urban and rural China (7–10), which may translate into inequalities in the economic status, educational opportunities, employment and health outcomes (11–13). Education—perhaps the most salient modifiable social determinant and an upstream cause of health, is a powerful means of reducing socioeconomic and political disadvantages, to achieve upward mobility (14). However, the dual social structure causes disparities in the acquisition and utilization of educational resources between urban and rural residents (15–17). For the post-90s generation, the probability of urban students attaining higher education is 90% higher than that of rural students (65.12 vs. 34.41%) (18). Thus, education can also reproduce social class and health inequality. Poor education in rural areas can directly reduce the happiness perception of rural residents and negatively impact their happiness perception through income and social class perception gap (19). In this case, clarifying the nexus and mechanism between education and health is critical to avoid the unintended consequence of aggravating class solidification, which is beneficial to urban-rural integrated development.

Previous research has demonstrated a significant relationship between educational achievement and multiple health consequences, including mortality, self-rated health status, morbidity using objective health measures such as blood pressure, body mass index, hypertension, and chronic disease, and health-related behaviors such as smoking and drinking (20–22). However, so far, there is no consensus on the relationship between education and health. Some studies have reported a positive effect (23, 24), while others have reported no or even negative effects (25–27). The health benefits of education may vary among people with different socioeconomic statuses in different stages of education (28, 29), and a non-linear association should exist between education and health. Whilst numerous studies have explored individuals' health—focusing on physiological and behavioral factors—family, as a systematical unit to develop multifaceted material, psychological, emotional, social, and cultural capital for health, has attracted less attention (30). Moreover, the social context in which education and health processes are embedded has been ignored, which may have a limited impact on addressing disparities (31). From the lens of structuralism (32), the economic position and living conditions determined by the social structure can cause health inequalities (33). Nowadays, topics such as “small-town swot,” “impoverished families can hardly nurture rich sons,” and “schooling is useless” are heatedly debated. Therefore, it is necessary to explore the health benefits of education in China's unique dual social context. The present study examines the impact of education on FH for urban and rural residents, which may provide a panoramic view for policymakers, educators, and health practitioners to conduct interventions aimed at specific populations to reduce social inequality and promote common prosperity.

## 2. Literature review and hypothesis

The family forms a the basic foundation for the individual and community health, as well as the basic unit of health care, disease prevention, and health promotion in the twenty-first century (34). Families are biologically, legally, or emotionally linked groupings, and health can “spread” through familial bonds. According to the family system theory, family members are interrelated, and individuals' health outcomes are determined by their family members (35).

Education can generate health externalities for individuals and their families through economic, health-behavior, and social-psychological paths (36) to preserve family wellbeing inter- and trans-generationally. Highly-educated people usually have life partners with similar educational backgrounds and professions, which can promote family harmony. Meanwhile, better-educated parents are more likely to live in a safe neighborhood and have stable family lives, providing sufficient material and spiritual support and developing healthy habits for their offspring (37, 38). In turn, better-educated adults can obtain decent jobs with higher salaries, relieving their parents' budget constraints, and making good use of health resources for physical examination and chronic disease management (39).

However, the long-term existence of uneven distribution of educational resources due to the Hukou system causes the segmentation of the labor market, the fragmentation of economic status, lifestyle, and social interaction, and the reproduction of health inequality (40). In the stage of compulsory education, rural schools are left behind in basic equipment, quality of instruction, attracting highly qualified teachers, peer influence, parental expectations, and extracurricular training (41, 42). As a result, the opportunity to attend high school is biased toward urban residents. Moreover, schools in rural areas lack a physical exercise curriculum, sports facilities, and health concepts, leading to low health literacy among rural students from an early age (43, 44). Although access to higher education has increased with the college enrollment expansion policy, there is still uneven distribution of higher education due to the impact of family background and magnified regional differences (45). According to the maximum maintenance inequality hypothesis and the effective maintenance inequality hypothesis (46, 47), the superior class will crowd out the educational opportunities of the inferior class, preventing the elimination of inequality until the educational opportunities are saturated for the superior class. Moreover, the type of inequality transforms from the simple quantity to the differences in quality of enrollment and level of the university. Constrained by economic conditions and cultural horizon, it is more difficult for rural residents to succeed in the college entrance examination (48), and they are even more likely to make a “rational” decision to give up their education. Therefore, hypothesis 1 is proposed based on the disparities between urban and rural China.

**Hypothesis 1 (H1):** Disparities in education cause FH inequality between urban and rural China.

Since the access to higher education is limited, highly competitive, and selective, rural students have to make greater efforts and overcome more barriers to secure college admission. The opportunity for urban residents to attend university was 3.4 times that of rural residents among those born between 1975 and 1979, which increased to 5.5 times among those born between 1980 and 1985 (50). Higher education attainment may not make rural students and their families happier because of the high cost, low rates of return, and a prolonged period of investment (51). Wang et al. demonstrated that the positive spillover effect of higher education is significant only in urban families (52). Moreover, after the expansion policy, the job market is flooded with college graduates (over 10 million), leading to the devaluation of diplomas and the mismatch with the employers' demand. Worse still, the labor market segmentation occasioned by the hukou system exposes graduates from rural areas to a higher risk of unemployment and low-wage employment.

Additionally, Grossman proposed that everyone obtains the initial health stock at birth through heredity, which is maintained or improved through later individual or public investment (53). There are disparities in health stock between urban and rural residents caused by endowments and social determinants. According to the “resource multiplication” or “add protection” theory (54, 55), education has multiplicative health benefits for an advantaged subpopulation. Urban residents have a preference for a healthy lifestyle, which can be solidified and reinforced through the process of education. However, it is more difficult for rural residents to obtain and translate educational gains into health benefits for the whole family due to the lack of a health concept and health resources (56). Moreover, accessing higher education is a crucial ladder for career development and social status for rural residents. While for urban residents with superior congenital conditions, higher education is more about cultural expectations and spiritual pursuit than just making a living. Thus, rural residents may suffer greater psychological deprivation and family-raising pressure when encountering negative events such as economic slumps and unemployment, which reduce their perception of happiness and family wellbeing (57). As a result, hypothesis 2 is proposed.

**Hypothesis 2 (H2):** Rural residents receive fewer FH benefits of higher education than urban residents.

According to the life course theory (49), the health benefits of education differ depending on the stage of education. When the educational level is relatively low, increasing years of schooling (YS) can greatly improve the health status; however, beyond a certain threshold, continued increase can hardly have a health premium. In western studies, although individuals with a secondary education diploma have the highest perception of happiness, the “marginal” health promotion “increment” is reduced after individuals obtain a college degree (58, 59). Highly-educated people tend to have higher expectations and are usually in a state of tension, anxiety, and disappointment, which may in turn offset the potential mental health benefits (60). For example, Avendano et al. found that increasing YS could incur psychological stress and emotional burdens (61). Even worse, these negative emotions are usually ignored or even suppressed, which increases the risk of unhappiness. In 2019, Nature investigated more than 6,300 doctoral students around the world and 36% of respondents had sought help because of anxiety or depression (62). Besides, the rate of sleep problems and suicide attempts has increased among Chinese college students from 2010 to 2020 (63). Hypothesis 3 is proposed that a non-linear relationship exists between education and health.

**Hypothesis 3 (H3):** There is an inverted U-shaped relationship between YS and FH.

The work-family conflict (WFC) occurs when demands and negative moods experienced in the work domain spill over into the family domain, which potentially undermines wellbeing, family functioning, and social relationships (64–69). WFC is significantly related to affective disorders including anxiety, depression, and suicidal ideation (70, 71). Frone et al. posited that mediates the relationship between work and family microsystems (65). Aryee et al. found that WFC mediates the effects of paid work and family systems on individual and family outcomes (72), including job and family satisfaction, psychological health (73), marital tension (74), and parenting (75). Highly-educated people are more engaged in administrative management, and professional or technical work under a greater cognitive load, leading to extensive exposure to electronic products, irregular diet and rest schedules, lack of exercise, depression, and chronic diseases (76). The work stressors and negative affect can cross over within families and ultimately lead to family dysfunction (77, 78). Therefore, hypothesis 4 is proposed.

**Hypothesis 4 (H4):** WFC can negatively mediate the relationship between education and FH.

## 3. Methods

### 3.1. Setting, sample, and data collection

We carried out a cross-sectional nationwide survey from July to September 2021 to collect data on trends in China's wellbeing for people, families, communities, and cities. A total of 120 cities were randomly chosen from 23 provinces, capitals of five autonomous regions, and four province-level municipalities using a multistage cluster sampling technique. In each city, at least one surveyor or survey team was hired. Each surveyor was tasked with gathering 30–90 questions, and each team was tasked with gathering 100–200 questionnaires. The enumerators collected a sample with gender, age, and urban/rural distribution that generally matched the demographics based on the results of the “7th National Census, 2021”. After removing respondents aged <18 years, the final sample included 9,964 responses [urban, *n* = 5,796 (58.2%); rural, *n* = 4,168 (41.8%)].

### 3.2. Measurement of key variables

#### 3.2.1. Dependent variable

Family health (FH), which served as the primary explanatory variable, composed of family social and emotional health process, family health lifestyle, family health resources, and family external social supports (Supplementary Table 1), and was measured by a 10-item abbreviated version of the Family Health Scale (FHS-SF) (30). FHS-SF with Cronbach's α of 0.849 demonstrates good validity and reliability. Five response levels from strongly disagree (1) to strongly agree (5) were used to calculate the score. Negatively worded items were reverse scored so that higher scores indicated better FH.

Self-reported health and health score—measured by the EQ-5D-5L questionnaire—were used for robustness tests. EQ-5D-5L was used to define and assess health in various illness categories (79). The EQ-5D-5L descriptive system is composed of five dimensions, mobility, self-care, usual activities, pain or discomfort, and anxiety or depression. Five response levels ranging from 1 to 5 for no problems, slight problems, moderate problems, severe problems, and unable to/extreme problems, respectively, were used to calculate the score.

#### 3.2.2. Independent variable

The primary explanatory variable was years of schooling (YS), with 0 denoting illiteracy, six denoting primary school, nine denoting junior high, 12 denoting high school, 15 denoting an associate's degree, 16 denoting a bachelor's degree, 19 denoting a master's degree, and 22 denoting a Ph.D. degree (80).

#### 3.2.3. Control variables

Age, gender, marital status, religion, household income, healthcare, siblings, number of children, homestyle, ethnicity, and job type were all considered as control variables for individuals and family factors. Marital status was classified as married or others (single/divorced/widowed). Religion was classified as religious or not. Household income represents monthly household income per capita. Healthcare was classified as either out-of-pocket or purchased. Traditional homestyle was defined as couple family, nuclear family, main family, and united family, while others were defined as non-traditional homestyle. Ethnicity was classified as Han or other ethnic groups. Ethnicity was classified as Han or other ethnic groups. Workers were classified into three types: first-type, including government workers, enterprise managers, and professionals; second-type, including clerks, businessmen, producers, operators, and military personnel; and third-type, including agricultural, forestry, animal husbandry, fishery, water conservancy production personnel.

#### 3.2.4. Mediating variable

WFC was measured through an index of five items (Supplementary Table 2). The items were answered on a five-point rating scale that ranged from strongly dissatisfied to strongly satisfied. The descriptive statistics for each of these variables are shown in Table 1.

**Table 1:** Descriptive statistics and univariate analysis.

**Variables**	**All**	**Urban**	**Rural**	***p***
Total	9,966	5,798 (58.2)	4,168 (41.8)	
Family health				0.000
Mean (SD)	3.81 (0.67)	3.86 (0.68)	3.75 (0.65)	
Self-reported health				0.000
Mean (SD)	81.49 (17.89)	82.34 (17.31)	80.30 (18.61)	
Health score				0.000
Mean (SD)	0.95 (0.12)	0.95 (0.11)	0.94 (0.13)	
Years of schooling				0.000
Mean (SD)	13.24 (4.45)	14.38 (3.70)	11.64 (4.88)	
Educational attainment n (%)				0.000
Pre-higher education	3,837 (38.5)	1,583 (27.3)	2,254 (54.1)	
Higher education	6,129 (61.5)	4,215 (72.7)	1,914 (45.9)	
Age				0.395
Mean (SD)	39.67 (15.49)	39.56 (14.45)	39.82 (16.83)	
Gender n (%)				0.172
Male	4,591 (46.1)	2,705 (46.7)	1,886 (45.2)	
Female	5,375 (53.9)	3,093 (53.3)	2,284 (54.8)	
Marital status *n* (%)				0.000
Single/divorced/widowed	3,740 (37.5)	1,982 (34.2)	1,758 (42.2)	
Married	6,226 (62.5)	3,816 (65.8)	2,410 (57.8)	
Ethnicity *n* (%)				0.000
Han	9,401 (94.3)	5,516 (95.1)	3,885 (93.2)	
Minority	565 (5.7)	282 (4.9)	283 (6.8)	
Religion *n* (%)				0.000
Infidelity	9,661 (96.9)	5,650 (97.4)	4,011 (96.2)	
Others	305 (3.1)	148 (2.6)	157 (3.8)	
Household income				0.000
Mean (SD)	4,642.69 (3,727.52)	5,515.62 (3,823.54)	3,428.38 (3,217.76)	
Healthcare *n* (%)				0.000
Self-paid	1,931 (19.4)	869 (15.0)	1,062 (25.5)	
Others	8,035 (80.6)	4,929 (85.0)	3,106 (74.5)	
Siblings *n* (%)				0.000
0	2,230 (22.4)	1,681 (29.0)	549 (13.2)	
≥1	7,736 (77.6)	4,117 (71.0)	3,619 (86.8)	
Number of children *n* (%)				0.000
0	4,002 (40.2)	2,247 (38.8)	1,755 (42.1)	
1	3,058 (30.7)	2,266 (39.1)	792 (19.0)	
2	2,231 (22.4)	1,076 (18.6)	1,155 (27.7)	
≥3	675 (6.8)	209 (3.6)	466 (11.2)	
Homestyle *n* (%)				0.786
Traditional	9,063 (90.9)	5,277 (91.0)	3,786 (90.8)	
Non-traditional	903 (9.1)	521 (9.0)	382 (9.2)	
Job type *n* (%)				0.000
First-type	1,551 (33.3)	1,246 (38.9)	305 (21.0)	
Second-type	1,750 (37.6)	1,133 (35.4)	617 (42.4)	
Third-type	1,358 (29.1)	825 (25.7)	533 (36.6)	
Work-family conflict				0.522
Mean (SD)	12.88 (4.53)	12.85 (4.49)	12.92 (4.59)	

χ^2^ tests for categorical variables and analysis of variance for continuous variables. Source: Own survey result, 2021. SD, standard deviation.

### 3.3. Data processing and statistical analysis

All data were analyzed using *R* statistical software version 4.1.2 (81). The eq5d package was used to calculate the health score (82). Oaxaca-Blinder decomposition for linear regression models was performed using the Oaxaca package (83). Propensity score matching (PSM) was conducted using the MatchIt package (84). Mediating effect was performed using the mediation package (85).

Stage 1. Univariate analysis and descriptive statistics were used. To determine whether there was a statistically significant difference in the variables between China's urban and rural areas, the *P*-value was provided. Categorical variables were compared using chi-square analysis. Continuous variables were compared using an independent *t*-test.

Stage 2. To estimate the impact of YS on FH in the urban and rural areas, the following regression model was built:


(1) $$\begin{array}{l} \text{F}{\text{H}}_{\text{i}}=\alpha_{0}+\alpha_{1}\text{YS}+\text{βX}+\mu_{\text{m}}+\varepsilon_{\text{i}} \end{array}$$

FH represents family health. YS represents years of schooling. X represents a set of control variables. μ represents fixed effect. ε represents a random perturbed variable. In all subscripts, i represents the individual and m represents the province.

Blinder-Oaxaca decomposition was developed and is commonly utilized in labor market discrimination research (86). Economists and sociologists, for example, have used it to break down income and earnings disparities depending on gender (87) and ethnicity (88). Although Blinder-Oaxaca decompositions have long been used in empirical studies on discrimination, they can be used to explain variations in any continuous outcome between any two groups. The decomposition divides the difference in mean outcomes into a portion that can be explained by cross-group differences in the explanatory factors and a portion that cannot. Discrimination has frequently been blamed for the unexplained fraction of the mean outcome gap.

A thorough comparison was conducted between urban and rural areas to test Hypothesis 1. The Oaxaca-Blinder (OB) model was used to determine how much of the variance in mean results between urban and rural areas was caused by group differences in the levels of explanatory variables and how much was caused by variations in the size of the regression coefficients (89). The urban-rural FH gap can be broken down into two main components, according to the (OB) model, which is a counterfactual approach based on the supposition that “rural individuals had the same attributes as their urban counterparts”.


(2) $${\bar{H}}_{u}-{\bar{H}}_{r}=\;\left({\bar{X}}_{u}^{\prime }-{\bar{X}}_{r}^{\prime }\right){\hat{\beta }}_{u}+{\bar{X}}_{u}^{\prime }\left({\hat{\beta }}_{u}-{\hat{\beta }}_{r}\right)$$

${\bar{\text{H}}}_{\text{u}}$ and ${\bar{\text{H}}}_{\text{r}}$are the FH status for the urban and rural areas; X is the explanatory variable; ${\hat{\beta }}_{u}$ and ${\hat{\beta }}_{\text{r}}$ indicate the coefficients of explanatory variables for the urban and rural areas, respectively. The endowment effect (explained effect) and the coefficient effect (unexplained effect) can be used to explain the urban-rural FH divide. The former shows the proportion that may be attributed to the various degrees of explanatory factors in urban and rural locations. The latter refers to the proportion that can be attributed to explanatory variables that affect FH differently in urban and rural settings. Bootstrap sampling was computed based on 1,000 iterations.

Stage 3. To evaluate the inverted U-shaped link between YS and FH (hypothesis 3), the following regression model was constructed:


(3) $$\begin{array}{l} \text{F}{\text{H}}_{\text{i}}=\alpha_{0}+\alpha_{1}\text{YS}+\alpha_{2}\text{Y}{\text{S}}^{2}+\text{βX}+\mu_{\text{m}}+\varepsilon_{\text{i}} \end{array}$$

YS^2^ represents the square of years of schooling. The model's other definitions are identical to those in Equation (1).

Stage 4. Robustness was tested using two approaches. PSM (90) was applied in the first approach. Dummy variables were created for YS. YS was separated into five groups, ranging in size from small to large. The first group received a value of 0, the fifth group received a value of 1, and the middle three groups were not utilized. The above-mentioned variables were used to build the model. Then, using the nearest neighbor matching technique (ratio = 1, caliper size = 0.05), the people in the two groups were matched according to their propensity score values. A balanced distribution of each covariate between the two groups after matching is necessary for the PSM conclusion to be considered reliable. Therefore, the difference in FH may be attributable to YS rather than potentially confounding factors.

A substitute dependent variable was used in the second approach. To ascertain if the patterns were consistent, health metrics such as self-reported health and health score as dependent variables were added.

Stage 5. To test hypothesis 4 that WFC is a significant mechanism by which YS affects FH, a mediating effect analysis method (91) was applied.


(4) $$\begin{array}{l} \text{WF}{\text{C}}_{\text{i}}=\alpha_{0}+\alpha_{1}\text{YS}+\text{βX}+\mu_{\text{m}}+\varepsilon_{\text{i}} \end{array}$$

(5) $$\begin{array}{l} \text{F}{\text{H}}_{\text{i}}=\alpha_{0}+\alpha_{1}\text{YS}+\alpha_{2}\text{WF}{\text{C}}_{\text{i}}+\text{βX}+\mu_{\text{m}}+\varepsilon_{\text{i}} \end{array}$$

WFC represents work-family conflict. The model's other definitions are identical to those in Equation (1). For regression coefficients, Sobel Goodman mediation tests were performed. Bootstrap sampling was computed based on 500 iterations.

## 4. Results

### 4.1. Descriptive results

Demographic characteristics of the total sample as well as the rural and urban subsamples are displayed in Table 1. The proportion of respondents from urban and rural areas in the whole sample was 58.2 and 41.8%, respectively. At a 1% level, a statistically significant difference was found between the urban and rural subsamples in FH, self-reported health, health score, YS, educational attainment, marital status, religion, household income, healthcare, siblings, number of children, ethnicity, and job type. The mean FH score was 3.86 and 3.75 in the urban and rural subsamples, respectively. The mean number of YS was 14.38 and 11.64 in the urban and rural subsamples, respectively, a difference of almost 3 years. There was a significant difference in the acquisition of higher education between the urban and rural subsets (72.7 vs. 45.9%). The disparity in the urban-rural monthly household income per capita was more than 2,000 RMB. Regarding healthcare, the proportion of self-paid residents in the urban subsample was much lower than that in the rural subsample (15.0 vs. 25.5%). The proportion of the first-type job in the urban subsample was nearly twice that in the rural subsample (38.9 vs. 21.0%). The proportion of the second-type job was 35.4 and 42.4% in the urban and rural subsamples, respectively. The proportion of the third-type job was higher in the rural subsample than in the urban subsample (36.6 vs. 25.7%).

### 4.2. Average FH benefits from YS in urban and rural China

The regression analysis of YS and FH is shown in Table 2. The interaction between YS and hukou was significant (β = 0.011, *p* < 0.001). Residents with urban hukou had a greater regression coefficient (β = 0.015 vs. β = 0.007) and significance level (*p* < 0.001 vs. *p* < 0.5) than residents with rural hukou, which were both significant in YS. Moreover, residents with urban hukou had better FH than their rural counterparts (β = 0.050, *p* < 0.001).

**Table 2:** Effects of YS on FH between urban and rural area.

	**(m1)**	**(m2)**	**(m3)**	**(m4)**
	**All**	**Interaction**	**Urban**	**Rural**
YS	0.010^***^	0.005^#^	0.015^***^	0.007^*^
	(0.002)	(0.002)	(0.003)	(0.003)
Hukou (ref: rural)	0.050^***^	−0.097^*^		
	(0.015)	(0.043)		
YS^*^ Hukou (ref: rural)		0.011^***^		
		(0.003)		
Age	−0.004	−0.004	−0.005	−0.002
	(0.003)	(0.003)	(0.004)	(0.004)
Age^2^	0.000^*^	0.000^*^	0.000^*^	0.000
	(0.000)	(0.000)	(0.000)	(0.000)
Gender (ref: female)	−0.103^***^	−0.102^***^	−0.104^***^	−0.097^***^
	(0.013)	(0.013)	(0.017)	(0.020)
Marital status (ref: single/divorced/widowed)	0.074^***^	0.073^***^	0.075^**^	0.064^*^
	(0.021)	(0.021)	(0.027)	(0.031)
Ethnicity (ref: rural)	0.049	0.052	0.061	0.058
	(0.031)	(0.031)	(0.043)	(0.044)
Religion(ref: others)	−0.049	−0.051	−0.040	−0.062
	(0.039)	(0.039)	(0.056)	(0.055)
Household income	0.000^***^	0.000^***^	0.000^***^	0.000
	(0.000)	(0.000)	(0.000)	(0.000)
Healthcare(ref: self-paid)	0.187^***^	0.187^***^	0.196^***^	0.176^***^
	(0.017)	(0.017)	(0.025)	(0.023)
Siblings	0.028	0.034	0.019	0.069^*^
	(0.017)	(0.017)	(0.022)	(0.031)
Number of children	0.011	0.007	−0.011	0.026
	(0.011)	(0.011)	(0.015)	(0.016)
Homestyle (ref: non-traditional)	0.095^***^	0.099^***^	0.093^**^	0.106^**^
	(0.024)	(0.024)	(0.032)	(0.036)
Fixed effect	Yes	Yes	Yes	Yes
*N*	9,966	9,966	5,798	4,168

Standardized regression coefficient, with standard errors in parentheses; ^#^*p* < 0.1; ^*^p < 0.05; ^**^p < 0.01; ^***^p < 0.001. YS, years of schooling; FH, family health. Fixed effect is the province fixed effect.

**Figure 1:**
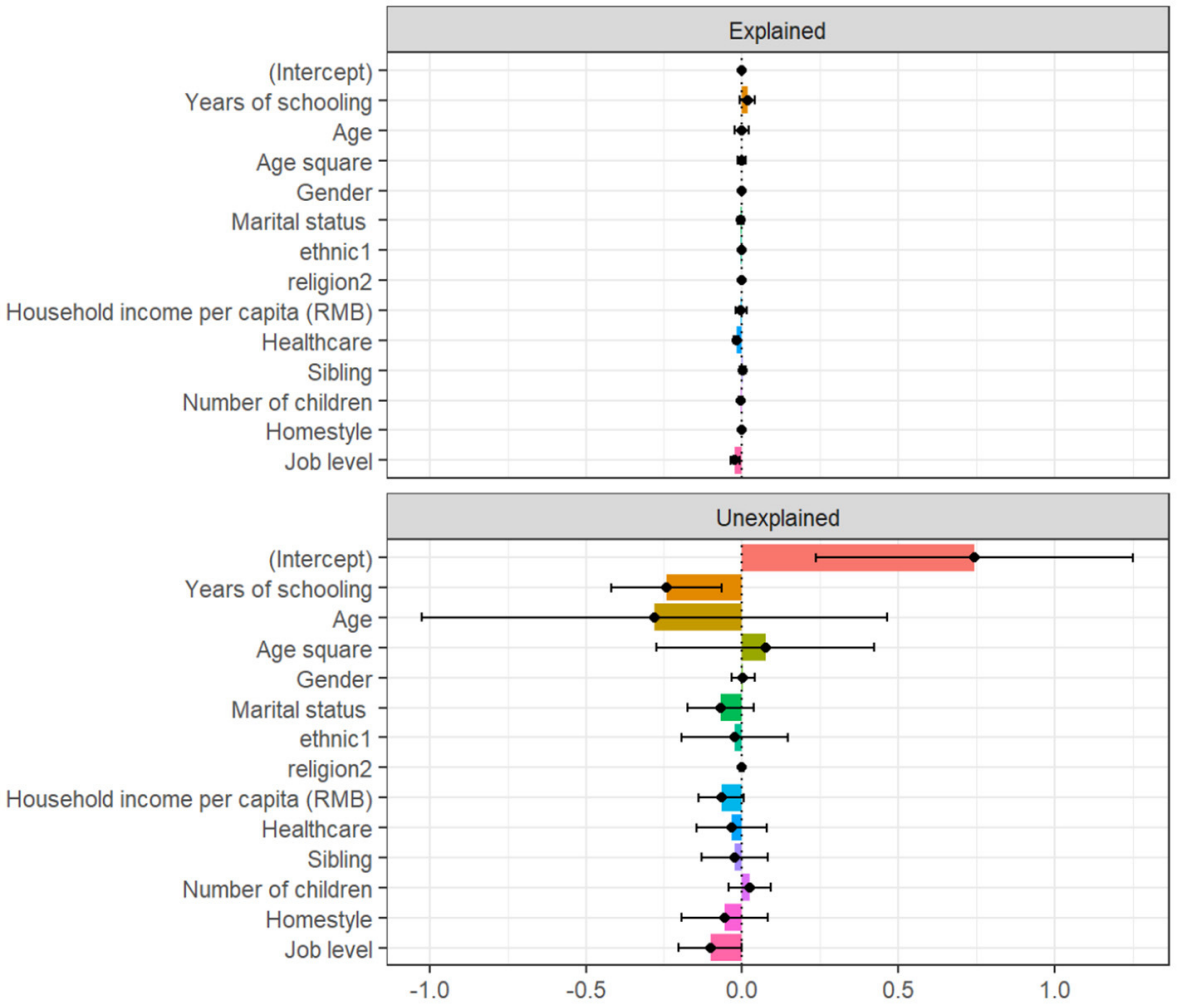
Oaxaca-Blinder decomposition of years of schooling between urban and rural subsamples.

Figure 1 and Table 3 displays the Oaxaca-Blinder results. Bootstrap sampling was performed with 1,000 iterations. For FH by hukou, the upper half was divided into endowment differences, and the lower half was separated into coefficient differences of variables. As shown in Supplementary Table 4, the total gap in FH between urban and rural residents was 0.089 (*p* < 0.001). The endowment effect was significant (Coef = 0.101, *p* < 0.001). YS accounted for 25.8% of the total FH gap. Specifically, if residents with rural hukou have the same YS as residents with urban hukou, then the FH gap would reduce by 0.023. It was evident from the coefficient section that there was a substantial difference in FH benefits of YS by hukou (Coef = 0.202, *p* < 0.05). This implies that the FH benefits of YS for residents with urban hukou are larger than those for residents with rural hukou, which is consistent with the previous regression results.

**Table 3:** Oaxaca-Blinder decomposition between urban and rural subsamples.

	**Overall**	**Explained**	**Unexplained**
		**Coef. (SE)**	**Coef. (SE)**
**FH**			
Urban	3.894^***^ (0.012)		
Rural	3.805^***^ (0.017)		
Difference	0.089^***^ (0.021)	0.101^***^ (0.012)	−0.012 (0.023)
YS		0.023^**^ (0.009)	0.202^***^ (0.074)
Age		0.011 (0.009)	0.270 (0.367)
Age^2^		−0.002 (0.004)	−0.072 (0.171)
Gender		−0.000 (0.001)	−0.004 (0.020)
Marital status		0.012^***^ (0.004)	0.060 (0.046)
Ethnicity		0.002 (0.001)	0.023 (0.091)
Religion		−0.000 (0.001)	0.002 (0.004)
Household income		0.022^***^ (0.005)	0.048^*^ (0.026)
Healthcare		0.022^***^ (0.005)	0.031 (0.056)
Siblings		−0.005 (0.003)	0.026 (0.056)
Number of children		0.007^**^ (0.003)	−0.027 (0.039)
Homestyle		0.001 (0.001)	0.056 (0.069)
Job type		0.007^*^ (0.004)	0.117^*^ (0.060)
Constant			−0.745^***^ (0.260)
Observations	4,659	4,659	4,659

Standard errors in parentheses. ^***^p < 0.01, ^**^p < 0.05, ^*^p < 0.1. FH, family health; YS, years of schooling; SE, standard error. The percentage of explained contribution: The Coef. of YS (0.023)/The overall urban-rural FH difference(0.089) = 25.84%.

### 4.3. Inverted U-shaped link between YS and FH

The inverted U-shaped relationship between YS and FH is shown in Table 4. Both urban and rural residents had substantial YS and YS^2^ values; the coefficient of YS was positive while that of YS^2^ is negative. The apogee of YS was about 17.8 (m5), whereas that in urban and rural areas was 17.1 (m6), and 13.7 (m7), respectively.

**Table 4:** The inverted U-shaped link between educational levels and FH.

	**(m5)**	**(m6)**	**(m7)**
	**All**	**Urban**	**Rural**
YS	0.119^***^	0.193^***^	0.109^**^
	(0.025)	(0.035)	(0.038)
YS^2^	−0.077^**^	−0.144^***^	−0.083^*^
	(0.025)	(0.035)	(0.039)
Control variables	Yes	Yes	Yes
Fixed effect	Yes	Yes	Yes
*N*	9,966	5,798	4,168

Standardized regression coefficient, with standard errors in parentheses; ^*^p < 0.05; ^**^p < 0.01; ^***^p < 0.001. The control variables are the same as the regressions in Table 2. Fixed effect is the province fixed effect. YS, years of schooling; FH, family health.

**Figure 2:**
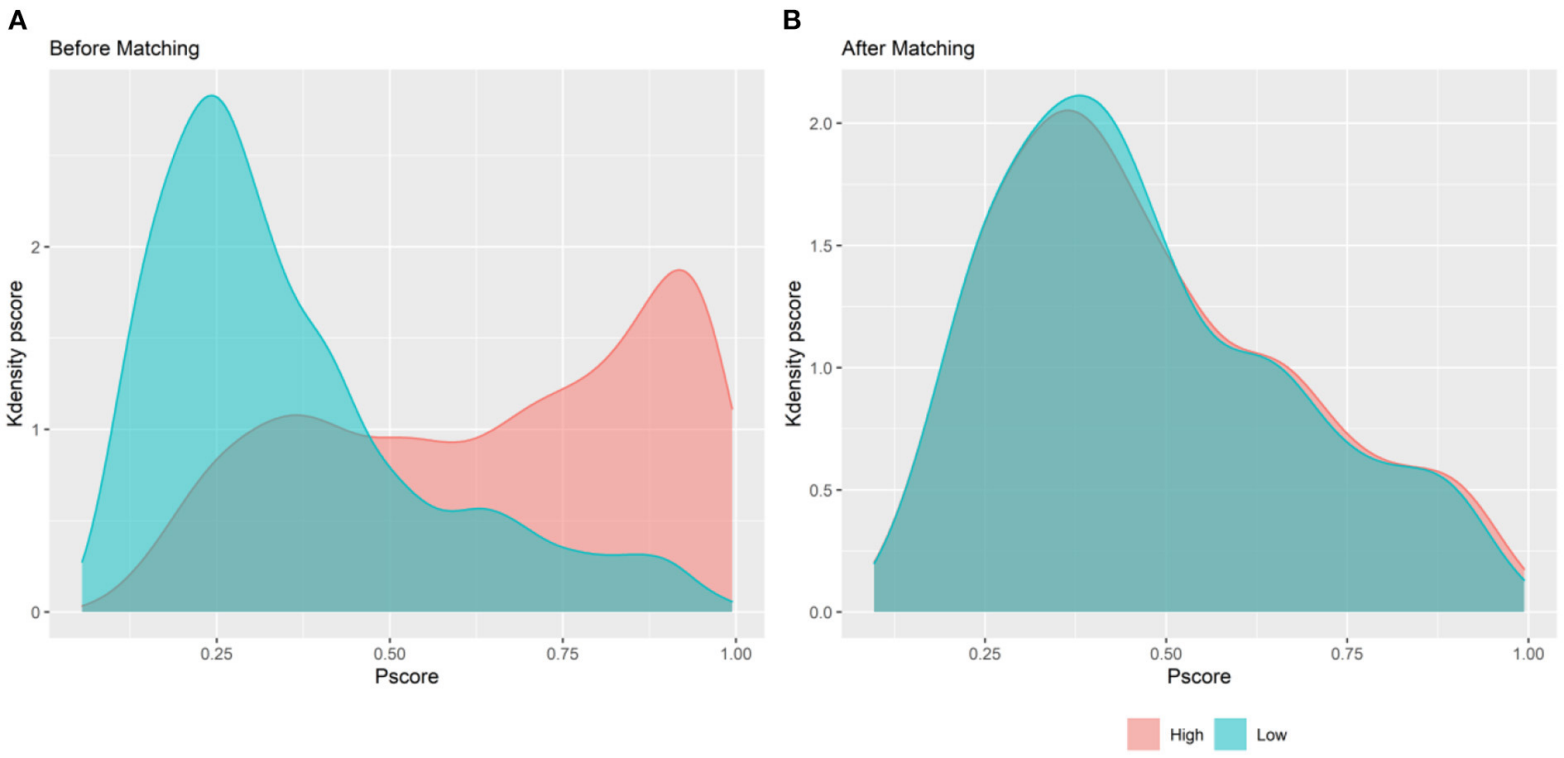
Kdensity distribution of propensity score. **(A)** Before PSM; **(B)** after PSM.

Dummy variables were established for YS as follows: the middle three groups were not used, the first group earned a value of 0, and the fifth group received a value of 1. The matching effect was confirmed by comparing the kernel density distributions of the first group and the fifth group before and after matching (Figure 2).

Reliability test results are shown in Table 5. According to the outcomes of regression analysis with the model (m8), the inverted U-shaped association between YS and FH remained significant after matching. Regression analysis with models (m9) and (m10) demonstrates that the results were still significant after changing the dependent variable.

**Table 5:** Effects of YS on HF, self-reported health, and health score.

	**(m8)**	**(m9)**	**(m10)**
	**PSM**	**Self-reported health**	**Health score**
YS	0.038^***^	0.752^***^	0.009^***^
	(0.009)	(0.156)	(0.001)
YS^2^	−0.001^**^	−0.019^**^	−0.000^***^
	(0.000)	(0.007)	(0.000)
Control variables	Yes	Yes	Yes
Fixed effect	Yes	Yes	Yes
*N*	2,104	9,966	9,966

Standardized regression coefficient, with standard errors in parentheses; ^**^p < 0.01; ^***^p < 0.001. The control variables are the same as the regressions in Table 2. Fixed effect is the province fixed effect. YS, years of schooling; FH, family health; PSM, propensity score matching.

### 4.4. Mediating effect of WFC

As shown in Table 6, YS significantly exacerbated WFC in (m11) (β = 0.208, *p* < 0.01). WFC negatively affected FH in (m12) (β = −0.113, *p* < 0.001). Further, Table 7 illustrates the mediating effect of WFC by Bootstrapping. Mediating analysis (m13) revealed a negative partial mediating effect (β = −0.094, *p* < 0.05).

**Table 6:** The link between YS, WFH, and FH.

	**(m11)**	**(m12)**
	**WFC**	**FH**
YS	0.208^**^	0.060^***^
	(0.074)	(0.011)
WFC		−0.113^***^
		(0.008)
Control variables	Yes	Yes
Fixed effect	Yes	Yes
*N*	6,810	6,810

Standardized regression coefficient, with standard errors in parentheses; ^**^p < 0.01; ^***^p < 0.001. The control variables are the same as the regressions in Table 2. YS, years of schooling; FH, family health; WFC, work-family conflict.

**Table 7:** Mediating effect analysis by Bootstrap.

	**Coef**.	**95% CI lower**	**95% CI upper**	**Control variables**
ACME	−0.001^**^	−0.002	−0.000	Yes
ADE	0.013^***^	0.008	0.017	Yes
Total effect	0.012^***^	0.007	0.016	Yes
Prop. mediated	−0.094^**^	−0.222	−0.024	Yes

This table reports the results of the mediating effect analysis by Bootstrap. We resampled the sample 500 times. The control variables are the same as regressions in Table 2. ^**^p < 0.01; ^***^p < 0.001. ACME, average causal mediation effects; ADE, average direct effects; total effect, stands for the total effect (direct + indirect); prop. mediated, the proportion of the effect of the independent variable on the dependent variable.

## 5. Discussion

To the best of our knowledge, this is the first study to examine the relationship between YS and FH using national representative data. Although several previous studies have explored the material returns of education, health benefits—as significant non-material returns of education—need more attention (92). Health is shaped by interaction with the family, community, and society. Instead of the individual-focused approach, the current study analyzed the family-centered health benefits of education, which can help release the positive externalities of education. Family is the basic “cell” of society in China, and FH forms the cornerstone of national health, which is a significant indicator for the implementation of health policy and allocation of social resources. Chinese people have strong family consciousness, and health-related knowledge and skills can be disseminated through kinship links, benefiting family members (93). Sharing information related to health care and disease prevention among families, especially during the COVID-19 pandemic, can build a culture of health, and foster family resilience and wellbeing (94–96).

In the process of building a moderately prosperous society, the principal contradiction between people's needs for a better life and unbalanced and inadequate development should be overcome. Besides medical factors, health status can be affected by social determinants to a certain extent, hence, health promotion should be extended to cultural, psychological, and social perspectives. By expanding from micro, middle, to macro levels, the study clarifies the internal relations among individuals' education, family health, and social structure, which is of great value to address systemic vulnerabilities, improve practices, and ensure more equitable education and health outcomes.

First, we found disparities in FH, educational attainment, household income, healthcare coverage, and job type between urban and rural China, and education inequality can translate into health inequality. There is uneven distribution and utilization of public resources, with low health awareness and inadequate medical security in rural China (80). Second, in general, education may have a positive effect on FH both for urban and rural residents. However, there is no simple linear relationship between education and health. Our study found an inverted-U relationship between YS and FH, which illustrates an upper limit of the “health dividend” of education. Increasing education beyond a certain threshold may not have health benefits (97). Higher education may negatively affect health. Previous studies demonstrated that people with higher education are more likely to be diagnosed with hypertension and psychological distress (98), and to drink more and exercise less (99). In the present study, health benefits declined at the turning point of 17.1 YS in the urban subsample, which occurred earlier in the rural subsample at 13.7. This suggested that rural residents, obtain limited FH benefits from higher education (52). One study suggested that minoritized racial groups generally experience poorer health and obtain fewer health benefits from education (100). Besides, the proportion of higher education is significantly lower among residents of rural hukou (101). It takes more effort for individuals and their families to attain higher education (102). However, they have more material expectations for higher education because of the long-term investment (103). Moreover, they have to overcome more risks to withstand the screening of the labor market (57). Through mechanism analysis, we discovered that highly-educated people face stronger WFC, which undermines the FH benefits of education. One compelling explanation may be that highly-educated people usually migrate to first-tier cities with rapid economic development and higher living standard, where the work intensity and competitiveness encroach on their time and energy devoted to their families (104, 105), thus, they are faced with difficulties such as family regulation and family health management. It is more challenging for rural residents to settle in first-tier cities due to the inherited disadvantage in endowments and resources (42).

The study indicates the health benefits of different educational stages and heterogeneity of the impacts of Hukou. Education can exert substantial, lasting, and wide-ranging health benefits by modifying health behaviors, enhancing healthy psychology, and strengthening social interactions (106). Therefore, policymakers, healthcare practitioners, and educators, should develop joint strategies to suppress the health disadvantages caused by social factors. Besides, the gradient upgrading of human capital should be encouraged in rural areas through the consolidation of compulsory education, the popularization of high school education, and the extension of higher education. Meanwhile, the social inequalities caused by education should also be negated. Our study supports the “resource multiplication” theory, the advantages of urban residents in cultural resource stock and the utilization efficiency further widens the urban-rural health inequality. This suggests that strategies to prevent vulnerable groups from falling into the happiness “trap” of education, that is, to pursue education and self-development at the cost of individual health and family happiness should be developed.

In this study, we only measured the YS by an individual rather than the whole family. Notably, educational attainment can be influenced by the family to some extent. Thus, although the robustness of the results was high based on PSM and the substitution of the dependent variable, the causal relationship between education and health cannot be concluded given the cross-sectional nature of the data analyzed in this study. Future studies should explore and compare the health benefits of different educational stages, such as compulsory education, high education, and associate, bachelor, and postgraduate education. It should be on that the data used in this study was collected during the pandemic, and thus whether the findings can be generalized to other contexts before or after the pandemic should be further explored. Moreover, the extent of work-family conflict faced by people of different genders and occupational types, and how it mediates the relationship between education and health need to be analyzed in future.

## 6. Conclusions

In summary, health development and promotion are embedded in the family unit and social structure. The present study contributes to family-centered health promotion and targeted interventions for urban and rural populations, respectively. Contrary to the intuition that education can promote social equity, this study reveals that uneven distribution and utilization of educational resources exacerbate health inequalities between urban and rural China. In addition, health dividend decreases after higher education. WFC is believed to be the negative mechanism of the education-FH nexus, which warns highly-educated people to avoid falling into the happiness “trap” of education and maintain a balance between work and family. However, this requires the joint efforts of the government, educational and health institutions, and the labor market to broaden externalities in education.

## Data availability statement

The raw data supporting the conclusions of this article will be made available by the authors, without undue reservation.

## Ethics statement

The studies involving human participants were reviewed and approved by Jinan University. The patients/participants provided their written informed consent to participate in this study.

## Author contributions

CJ and YL: conceptualization and methodology. CJ: software. YL: writing—original draft preparation. XLu and XLi: writing—review and editing. WZ and YW: supervision and project administration. All authors have read and agreed to the published version of the manuscript.
