# Supplementary material for: Inverted U-shaped relationship between education and family health: The urban-rural gap in Chinese dual society
Source: Front Public Health. 2023 Jan 11;10:1071245. doi: 10.3389/fpubh.2022.1071245 (PMC9874332; doi:10.3389/fpubh.2022.1071245)
Supplement: Supplementary file 1 [file Data_Sheet_1.docx]

Supplementary Material

# Supplementary Tables

**Supplementary Table 1.** Factor Loading of Items Using the Exploratory Factor Analysis and Cronbach $\alpha$ of FHS-SF

| Items |
| --- |
| We support each other. |
| I feel safe in my family relationships. |
| We stay hopeful even in difficult times. |
| We help each other in seeking health care services when needed (such as making doctor’s appointments). |
| We help each other make healthy changes. |
| We do not trust doctors and other health professionals (R). |
| My family did not have enough money at the end of the month after bills were paid (R). |
| My family did not have adequate housing (R). |
| We have people outside of our family we can turn to when we have problems at school or work. |
| If we needed financial help, we have people outside of our family we could turn to for a loan (e.g., for 1000 CNY) |
| Cronbach’s 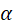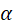 = 0.849 |

**Supplementary Table 2.** Factor Loading of Items Using the Exploratory Factor Analysis and Cronbach $\alpha$ of WFC

| Items |
| --- |
| My job takes time from me that I would like to spend with my family |
| After work, I am too tired when I come home to do some of the things I’d like to do (e.g., household and entertainment) |
| I miss a lot of time with my family because of my work |
| My work has a negative impact on my family life |
| Problems at work make me irritable at home |
| Cronbach’s 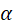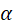 = 0.910 |
